# Effects of an action research-based nursing program on rheumatoid arthritis patients on long-term hormone therapy: A randomized controlled trial

**DOI:** 10.1097/MD.0000000000048643

**Published:** 2026-05-08

**Authors:** Lin Sun, Yue Zhang, Hai Huang, Chunfang Gu

**Affiliations:** a Department of General Medicine, Yancheng No.1 People’s Hospital, Affiliated Hospital of Medical School, Nanjing University, Yancheng, China; b Department of Rheumatology and Immunology, Yancheng No.1 People’s Hospital, Affiliated Hospital of Medical School, Nanjing University, Yancheng, China.

**Keywords:** action research, anxiety and depression, hormone therapy, rheumatoid arthritis

## Abstract

This study aims to evaluate the impact of an action research-based nursing program on patients with rheumatoid arthritis (RA) undergoing long-term hormone therapy. Eighty RA patients admitted to the Department of Rheumatology and Immunology from January 1, 2023, to May 31, 2024, and receiving long-term hormone therapy were recruited. Patients were randomized into 2 groups of 40 each: a control group receiving conventional care and an intervention group receiving care based on the action research method. Evaluations included daily measurements of fasting and 2-hour postprandial blood glucose levels, compliance assessed via a self-developed compliance scale with established reliability and validity, pain via a Visual Analog Scale, joint activity using the 28-joint Disease Activity Score, and anxiety and depression levels using self-rating scales. Baseline measurements showed no significant differences between the groups in fasting blood glucose, 2-hour postprandial blood glucose, pain, joint mobility, and anxiety and depression scores (*P* > .05). Post-intervention, both groups exhibited improvements in compliance, pain, joint mobility, and anxiety and depression scores, with the intervention group showing significantly greater improvements. The intervention group showed significantly smaller increases in fasting and 2-hour postprandial blood glucose than the control group. The action research-based nursing program significantly enhances compliance and joint mobility, controls blood glucose increases, and reduces pain, anxiety, and depression in RA patients on long-term hormone therapy. This method provides a practical reference for clinical nursing intervention in such patients.

## 1. Introduction

Rheumatoid arthritis (RA) is a chronic autoimmune disease characterized by progressive joint damage and systemic involvement. The disease trajectory is prolonged, marked by frequent relapses and a high incidence of disability.^[1]^ The prevalence of RA is escalating globally, with a notable increase in incidence correlating with advancing age, thereby significantly impairing patients’ quality of life and imposing considerable economic burdens on healthcare systems.^[2]^ Clinically, RA manifests with joint lesions, morning stiffness, swelling, and pain, accompanied by systemic complications such as mild to moderate anemia, osteoporosis, superficial lymphadenopathy, and hepatic and renal dysfunction. Prolonged disease progression often results in joint deformities and functional incapacitation, ultimately compromising patients’ workability and, in severe cases, posing life-threatening risks. Despite advancements in diagnostic and therapeutic modalities, the pathogenesis of RA remains incompletely understood, and current pharmacological interventions primarily aim to mitigate symptoms and control disease progression rather than providing a definitive cure. Glucocorticoids have been demonstrated to effectively suppress inflammation, alleviate joint pain, swelling, and stiffness, and inhibit bone destruction, thus being pivotal in RA management.^[3]^ Studies have shown that combined regimens of glucocorticoids and antirheumatic drugs enhance therapeutic efficacy, attenuate inflammatory markers, and improve clinical symptoms.^[4]^ However, the extensive and multifaceted actions of glucocorticoids are associated with significant adverse effects, including hyperglycemia, hypertension, osteoporosis, and aseptic bone necrosis, with long-term use notably increasing the risk of diabetes by 36% to 61%.^[5]^ Moreover, the psychological burden associated with prolonged glucocorticoid therapy often leads to anxiety, depression, and poor patient compliance. Thus, optimizing long-term therapeutic strategies to improve compliance, manage adverse effects, and address psychological well-being remains a critical focus of contemporary research in RA treatment.

Action research is a participatory research methodology involving collaboration between researchers and practitioners to address a specific problem. This approach leverages the combined expertise of both parties, facilitating a joint problem-solving process. Grounded in critical theory, action research employs an iterative cycle of problem identification, planning, action, observation, reflection, and replanning to implement dynamic interventions and quality improvements in practice settings.^[6,7]^ In nursing, Webb defines action research as “a small-scale intervention in real-world activities followed by a thorough examination of the intervention outcomes.”^[8]^ This methodology intimately links the resolution of practical workplace issues with scholarly research, transforming practitioners into active participants who drive the problem-solving process through continuous cycles of planning, action, observation, and reflection.

The literature reveals extensive applications of action research globally, encompassing domains such as nursing research,^[9]^ nursing education,^[10]^ and nursing practice,^[11]^ all demonstrating favorable outcomes. For instance, Johannessen utilized the iterative nature of action research to design essential oil aromatherapy interventions to improve sleep quality in Alzheimer patients, resulting in marked enhancements in their sleep.^[12]^ Similarly, Noguchhi et al^[13]^ implemented action research in an intensive care unit, enabling nurses to adopt a patient-centered perspective, enhancing communication with lightly sedated, mechanically ventilated patients, thereby boosting patient satisfaction and transforming ICU nursing practices. Maindal et al^[14]^ applied action research to early diabetes management, devising individualized lifestyle interventions for diabetic patients.

These studies confirm that action research, characterized by its problem-oriented approach, cyclic intervention structure, and active practitioner involvement, can precisely address practical clinical issues, making it particularly suitable for chronic disease management requiring long-term, multidimensional interventions. However, patients with RA on long-term hormone therapy face multiple challenges, including poor treatment compliance, difficulty in blood glucose control, psychological distress, and low adherence to rehabilitation exercises, necessitating a dynamic, multidisciplinary, and patient-engaged nursing model. Despite this, no study has applied action research to this specific population to date.

Therefore, this study introduces a nursing program based on action research tailored for RA patients undergoing prolonged hormone therapy. The program aims to enhance patient compliance and joint mobility, regulate blood glucose levels, and alleviate pain, anxiety, and depression. The detailed report follows.

## 2. Objects and methods

### 2.1. Study subjects

Patients with RA who were admitted to the Department of Rheumatology and Immunology of our hospital from January 1, 2023, to May 31, 2024, and were receiving long-term hormone therapy were selected as research subjects. Inclusion criteria: all RA patients were diagnosed according to the American College of Rheumatology and European League Against Rheumatism (EULAR) 2010 RA classification diagnostic criteria (American College of Rheumatology/EULAR 2010); those aged >18 years; those who had been on hormone therapy for >1 month; those with normal cognitive function; those with no other musculoskeletal conditions; those who agreed to participate in this study. Exclusion criteria: those who had cognitive or dyslexia, mental illness, etc, and could not effectively understand the content of the questionnaire; those who suffered from other connective tissue diseases; those who were planning to undergo surgery. The sample size was calculated according to the formula of comparing the means of the 2 samples, n1 = n2=([Zɑ/2 + Zβ] × σ/δ)², taking ɑ = 0.05, β = 0.1, and the two-sided test, and the table was obtained to obtain Zɑ/2 = 1.96, Zβ = 1.282. After consulting the relevant literature, we took σ=4.02, δ = 3.49. Substituting into the formula, we calculated n1 = n2 = 30, and the sample size should be 60 cases, but considering the 10% to 15% loss to follow-up, the sample size was finally determined to be 80 cases. Two independent researchers generated a random allocation sequence using a random number table. Patients were assigned sequentially upon enrollment to either the intervention group or the control group based on odd or even numbers, 40 cases per group. Allocation concealment was ensured by using sealed, opaque envelopes, each marked with a unique enrollment number, stored by a researcher not involved in the study. Envelopes were opened on-site at the time of patient enrollment to reveal group assignment, ensuring randomization and blinding of the allocation process. This study was approved by the Ethics Committee of Yancheng No.1 People’s Hospital (approval No. 2024-K-186). All patients provided written informed consent prior to participation.

### 2.2. Methods

The control group received a routine nursing intervention program, including the following: actively communicating with patients, understanding the results of various examinations of patients, evaluating the physical condition of patients, and treating symptoms; conducting health education for patients, explaining the pathogenesis, clinical manifestations, common drugs, precautions, etc of RA and improve patients’ understanding of the disease; closely monitor changes in patients’ blood glucose and blood pressure, guide patients to take medication according to the doctor’s advice, give patients relevant advice on daily diet and exercise, and encourage patients to maintain an optimistic attitude; correct patients’ poor posture to avoid postural deformity; introduce patients to hormone medication counseling in detail, including the mechanism of action, use and purpose of hormone medication, side effects, etc, to relieve patients’ anxiety, to allay patients’ concerns.

Based on the control group, the intervention group formed a multidisciplinary team including doctors, nurses, pharmacists, psychological counselors, nutritional therapists, and rehabilitation therapists, and formulated a care plan according to the basic steps of the action research method (plan-action-observation-reflection). The specific procedure is as follows.

#### 2.2.1. Composition of the research team

A multidisciplinary team was established under the leadership of the head nurses of the endocrinology and rheumatology departments. The research team included 1 physician from the endocrinology and rheumatology departments, 3 nurses from the rheumatology and endocrinology departments, 2 rehabilitation therapists, 1 pharmacist, 1 psychological counselor, and 1 nutritionist. The head nurses of the endocrinology and rheumatology departments were the project leaders, responsible for the research design and the organization and coordination of the entire project, as well as for monitoring the progress of the project and supervising the implementation of the measures; the endocrinologists and rheumatologists were responsible for the training of specialist knowledge such as the pathogenesis, clinical manifestations, commonly used drugs and precautions in RA; pharmacists were responsible for hormone medication management; psychological counselors were responsible for psychological counseling of patients; nutritionists were responsible for nutritional counseling of patients; endocrinology nurses were responsible for plan implementation and data collection, etc. Before the formal start of the study, all researchers received uniform training on action research steps and related knowledge, and they could only participate in this study after passing the assessment.

#### 2.2.2. Using the intervention method

According to the 4 basic steps of action research, 3 rounds of nursing intervention were implemented in combination with different stages of the patient’s illness (acute attack, remission, and stable stage). The 4 basic steps are planning, action, observation, and reflection. After each step is completed, the next round of 4 steps can be started. The specific plan is shown in Table 1.

**Table 1:** Implementation plan for the 3-round action research method.

Time	Objectives	Steps	Measures
The first acute attack	Teach patients how to do symptom management and rehabilitation exercises, control blood sugar, relieve pain and discomfort, reduce anxiety and depression, and encourage patients to do rehabilitation exercises.	Plan	Formulate a plan for this round: carry out targeted symptom management: including blood sugar management, pain management, etc; guide patients to carry out rehabilitation exercises; relieve patients’ anxiety and depression.
		Action	Implement various measures according to the plan: layered symptom management: blood glucose management: focus on blood glucose management. After admission, monitor the patient’s blood glucose, including fasting blood glucose and blood glucose 2 hours after meals. Record the blood glucose level to form the patient’s 24-hour blood glucose fluctuation curve. According to the blood glucose fluctuation curve, provide stepwise intervention and individualized management according to the doctor’s advice. Pain management. Educate patients about pain-related content, regularly use the VAS to assess the patient’s pain, clarify the location, degree, and type of pain, and give symptomatic treatment according to the patient’s pain degree. Instruct patients to perform rehabilitation exercises. Guide patients to do water resistance exercises, gradually increase the intensity and time of the exercise according to the patient’s muscle strength and do what the patient can bear. Emotional counseling for patients: nurses actively communicate with patients, understand the patient’s psychological status, analyze the patient’s existing psychological problems, and develop targeted psychological care plans based on the patient’s psychological characteristics.
		Observe	Through the implementation of symptom management and targeted one-to-one counseling, it was found that patients lacked knowledge about RA and were irregular in their use of medications, especially hormone medications.
		Reflect	For the problems in this round, select patients or their families to discuss solutions to problems that arise during the process and apply the solutions to the next round.
Second round of disease remission	Enable patients to acquire relevant knowledge about RA, increase their health awareness, and lay the foundation for them to take their medication regularly as prescribed by their doctor after discharge from the hospital.	Revised plan	The current round of plans: multidisciplinary team members will compile a “Health Manual for Rheumatoid Arthritis Patients” based on the research objectives and content. The content of the handbook should be as comprehensive and easy to understand as possible, including the etiology, incidence, clinical manifestations, treatment methods, medication instructions, blood sugar management, pain management, rehabilitation exercises, etc of RA, with emphasis on the poor treatment compliance of RA patients, the harm of anxiety and depression, and the importance of rehabilitation exercises; hold special lectures; provide targeted one-on-one counseling.
		Action	All measures were implemented according to the plan: after the patient’s symptoms were under control, the “Health Handbook for Rheumatoid Arthritis Patients” was distributed to help patients better understand the disease; special lectures on RA knowledge were held in the Endocrinology Department’s demonstration room every Tuesday and Friday afternoon using the “one lesson, two lectures” method to improve patients’ health awareness. Each patient was required to attend at least once a week; the special topics mainly included guidance on hormone use, stratified management of blood glucose, the importance of rehabilitation exercises, etc; one-on-one bedside education was conducted every Saturday morning to provide personalized guidance to patients and improve their cognition.
		Observation	Through ongoing discussions with patients and conscious testing of their understanding of disease-related knowledge, we found that patients did not understand the need for long-term hormone treatment after discharge from the hospital for RA, were concerned about the side effects of hormone treatment, and were unwilling to participate in rehabilitation exercises.
		Reflection	The second round of action research has solved the problems that the patients encountered in the first round, but it has also revealed other problems. For the problems revealed in this round, we will continue to select patients or their families to discuss solutions to the problems that arose during the auction process and apply the solutions to the next round of cycles.
The third round of stable disease	Eliminate concerns about the side effects of hormone therapy and allow patients to receive long-term hormone therapy and rehabilitation exercises after discharge.	Revise the plan again.	The current round of plans: nursing staff should emphasize the role of family members and guide them to help and supervise patients on long-term hormone treatment; hold special lectures on the importance of hormone drugs in RA treatment and the importance of rehabilitation exercises; set up a WeChat communication group for patients on the ward and set up a public WeChat account for the ward.
		Action	Implement various measures as planned: hold a symposium with the patient’s families. Continue the “one lesson, two lectures” approach from the previous round. Regularly send RA medication instructions, rehabilitation exercises, and other knowledge to WeChat public accounts and WeChat groups to remind and supervise patients; at the same time, invite patients with significant effects of long-term hormone treatment as role models to share their experiences in the group, allay patients’ fears and improve their compliance with long-term regular medication outside the hospital.
		Observation	After each knowledge lecture, patients or their families were asked questions about disease-related knowledge, and it was found that their knowledge and understanding of disease-related knowledge significantly improved; by observing patients’ behavior, it was found that patients were able to perform appropriate rehabilitation exercises and their compliance with regular medication improved.
		Reflection	After 3 rounds of nursing intervention, most patients were able to take their medication regularly and participate in appropriate rehabilitation exercises, and their anxiety and depression were reduced. This trial has been completed.

RA = rheumatoid arthritis, VAS = Visual Analogue Scale.

### 2.3. Monitoring indicators

(1) Compliance: The treatment compliance scale was developed by the research team based on a literature review and 2 rounds of expert consultation. It consists of 3 dimensions: medication compliance (7 items), dietary compliance (7 items), and rehabilitation exercise compliance (6 items), totaling 20 items. Each item is scored on a 5-point Likert scale (1 = never, 2 = rarely, 3 = sometimes, 4 = often, 5 = always), with total scores ranging from 20 to 100. Higher scores indicate better compliance. The scale was evaluated by 6 experts in rheumatology, endocrinology, nursing management, and psychology; the scale-level content validity index was 0.93, and the item-level content validity index for all items exceeded 0.83. In a pilot test involving 30 RA patients, the Cronbach α coefficient for the overall scale was 0.89, with subscale coefficients of 0.86, 0.88, and 0.84, respectively, indicating good internal consistency reliability.
(2) Blood glucose: Fasting blood glucose and 2-hour blood glucose after 3 meals were measured and recorded at 06:00 before and after the intervention using a blood glucose meter from the same manufacturer.
(3) Pain score: The visual analog pain scale was used to assess the pain symptoms of the 2 groups of patients before and after the intervention. The Visual Analog Scale (VAS) scoring method was first proposed by Hayes et al.^[15]^ The score range is 0 to 10 points, where 0 points indicate no pain symptoms and 10 points indicate severe pain symptoms. The higher the score, the more severe the patient’s pain symptoms.
(4) Joint mobility: The Disease Activity Score with 28-joint counts (DAS28) was used to assess joint mobility in the 2 groups before and after the intervention. The DAS28 scoring system was developed by the EULAR^[16]^ and includes tender joint counts, swollen joint counts, erythrocyte sedimentation rate, and patient global assessment. The lower the DAS28 score, the better the joint mobility.
(5) Anxiety and depression scores: Before and after the intervention, the Self-Rating Anxiety Scale (SAS) was used to assess the anxiety level of the 2 groups of patients, and the Self-Rating Depression Scale (SDS) was used to assess the depression level. The SAS and SDS each contain 20 items, each scored from 1 to 4. The higher the score, the more severe the patient’s anxiety and depression.

### 2.4. Data collection and processing

Data collection was conducted by the researchers themselves, with measurements taken at 2 time points: before intervention (upon admission) and after 4 weeks of intervention. Data included fasting blood glucose, 2-hour postprandial blood glucose, treatment compliance scale scores, VAS for pain, DAS28, SAS, and SDS. All completed questionnaires and scales were collected immediately after administration and checked for completeness and accuracy to prevent missing data or errors. Data were double-entered and validated before statistical analysis using computer software.

### 2.5. Statistical analysis

SPSS 22.0 statistical software was used to perform statistical analysis on the data. Normality of data distribution was assessed using the Shapiro–Wilk test. Measurement data that conformed to normal distribution were expressed as mean ± standard deviation, and the *t* test was used; specifically, the independent samples *t* test was used for between-group comparisons, and the paired *t* test was used for within-group comparisons before and after intervention. For the multiple daily postprandial blood glucose measurements, the average of the 3 post-meal values was calculated for each patient and used as a single observation in the analysis to avoid pseudoreplication. Count data were expressed as the number of cases and percentages, and the *χ*^2^ test was used. *P* < .05 indicated that the difference was statistically significant.

## 3. Results

**Figure 1.**
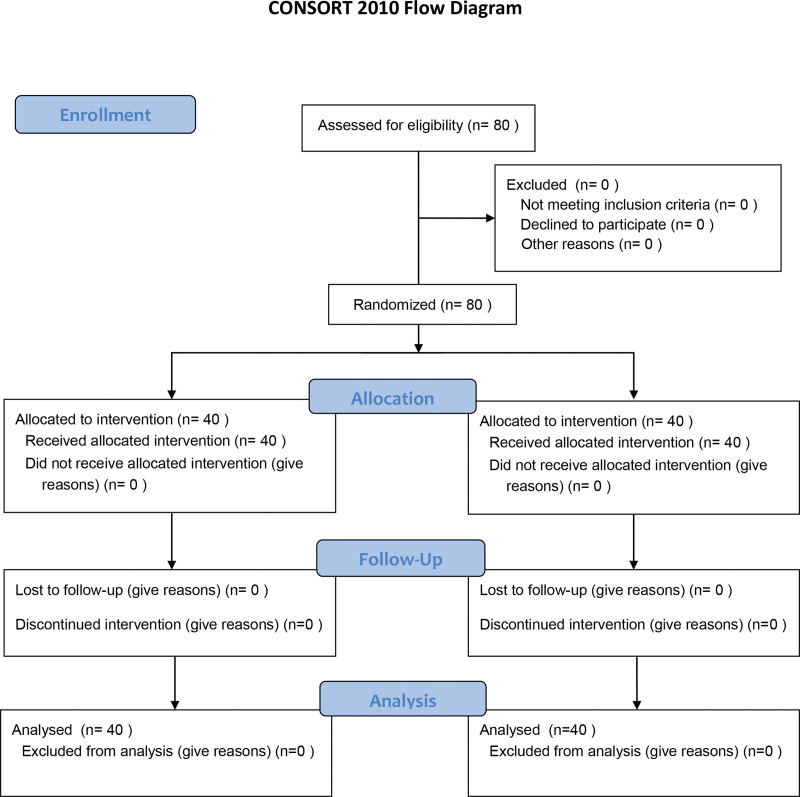
CONSORT flow diagram.

A total of 80 patients were enrolled and randomized into 2 groups of 40 each, with no loss to follow-up (Fig. 1).

### 3.1. Comparison of general information between the 2 groups of patients

There was no significant difference in the general data of the 2 groups of patients, such as sex, age, level of education, course of disease, and length of hospital stay (*P* > .05), indicating that the 2 groups were comparable. See Table 2 for details.

**Table 2:** Comparison of general information between the 2 groups of patients.

Item	Classification	Control group	Intervention group	*t*/*χ*^2^ value	*P* value
Sex	Male	24 (60.0%)	22 (57.1%)	0.059	.808
	Female	16 (40.0%)	18 (42.9%)	–	–
Age (yr)	–	61.58 ± 6.95	62.58 ± 5.79	−0.875	.384
Education	Junior high school and below	22 (51.4%)	23 (57.1%)	0.230	.631
	High school and above	18 (48.6%)	17 (42.9%)	–	–
Duration of illness (yr)	–	6.23 ± 4.81	5.98 ± 5.63	0.873	.385
Length of hospitalization (d)	–	15.35 ± 2.81	14.96 ± 3.15	0.871	.391

### 3.2. Comparison of compliance between the 2 groups of patients

After the intervention, patients in the intervention group had higher compliance with medication, diet, and rehabilitation than those in the control group, and the differences were statistically significant (*P* < .05), as shown in Table 3.

**Table 3:** Comparison of treatment compliance between the 2 groups after intervention (x̄ ± s, points).

Group	n	Medication compliance	Dietary compliance	Rehabilitation compliance	Total score
Intervention group	40	28.15 ± 1.58	29.24 ± 2.19	23.96 ± 1.49	81.35 ± 3.09
Control group	40	18.32 ± 1.82	18.78 ± 2.55	14.62 ± 2.44	51.72 ± 3.97
*t* value		25.871	19.563	20.347	37.092
*P* value		<.001	<.001	<.001	<.001

Between-group comparisons were performed using independent samples *t* test.

### 3.3. Comparison of fasting and 2-hour post-meal blood glucose between the 2 groups of patients

At baseline, there was no statistical difference in fasting blood glucose and 2-hour post-meal blood glucose between the 2 groups of patients (*P* > .05). After the intervention, the increase in fasting blood glucose and 2-hour postprandial blood glucose was lower in the intervention group than in the control group, and the difference was statistically significant (*P* < .05). See Table 4 for details.

**Table 4:** Comparison of fasting and 2-hour postprandial blood glucose between the 2 groups before and after intervention (x̄ ± s, mmol/L).

Group	n	Fasting blood glucose		2-hour postprandial blood glucose	
		Pre-intervention	Post-intervention	Pre-intervention	Post-intervention
Intervention group	40	5.36 ± 1.42	5.75 ± 2.69	10.42 ± 2.38	10.52 ± 1.36
Control group	40	5.23 ± 1.56	7.18 ± 3.17*	10.80 ± 2.61	14.97 ± 1.72*
*t* value (between)		0.591	−7.135	−0.589	−8.243
*P* value (between)		.738	.001	.759	<.001

Within-group comparisons (paired *t* test) showed a statistically significant increase in both fasting and 2-hour postprandial blood glucose in the control group after intervention (*P* < .05), whereas the increases in the intervention group were not statistically significant (*P* > .05). Between-group comparisons were performed using independent samples *t* test.

* *P* < .05.

### 3.4. Comparison of pain scores and joint range of motion scores between the 2 groups

At baseline, there was no statistical difference in pain scores (VAS) and joint range of motion scores (DAS28) between the 2 groups (*P* > .05). After the intervention, the VAS and DAS28 scores of both groups decreased, and the intervention group was lower than the control group, with a statistically significant difference (*P* < .05); the increase in fasting blood glucose in the intervention group was lower than that in the control group, and the difference was also statistically significant (*P* < .05), as shown in Table 5.

**Table 5:** Comparison of VAS and DAS28 scores between the 2 groups before and after intervention (x̄ ± s, points).

Group	n	VAS score		DAS28 score	
		Pre-intervention	Post-intervention	Pre-intervention	Post-intervention
Intervention group	40	5.35 ± 2.15	1.38 ± 0.56*	6.09 ± 1.21	3.49 ± 0.98*
Control group	40	5.19 ± 1.84	2.98 ± 0.92*	5.89 ± 0.98	4.05 ± 1.02*
*t* value		0.592	−10.328	0.412	−6.651
*P* value		.735	.001	.659	.003

Within-group comparisons (paired *t* test) showed significant reductions in both VAS and DAS28 scores in both groups after intervention (*P* < .05). Between-group comparisons were performed using independent samples *t* test.

DAS28 = 28-joint Disease Activity Score, VAS = Visual Analog Scale.

* *P* < .05.

### 3.5. Comparison of anxiety and depression scores between the 2 patient groups

At baseline, there was no statistical difference in the anxiety (SAS) and depression (SDS) scores between the 2 groups of patients (*P* > .05). After the intervention, the SAS and SDS scores of the 2 groups of patients decreased, and the intervention group was lower than the control group, with a statistically significant difference (*P* < .05). See Table 6 for details.

**Table 6:** Comparison of SAS and SDS scores between the 2 groups before and after intervention (x̄ ± s, points).

Group	n	SAS score		SDS score	
		Pre-intervention	Post-intervention	Pre-intervention	Post-intervention
Intervention group	40	69.59 ± 7.15	49.57 ± 7.54*	71.59 ± 8.15	47.52 ± 6.98*
Control group	40	68.91 ± 6.91	55.41 ± 6.98*	69.94 ± 7.98	54.95 ± 7.07*
*t* value		0.718	−8.421	0.705	−9.357
*P* value		.635	.001	.668	<.001

Within-group comparisons (paired *t* test) showed significant reductions in both SAS and SDS scores in both groups after intervention (*P* < .05). Between-group comparisons were performed using independent samples *t* test.

SAS = Self-Rating Anxiety Scale, SDS = Self-Rating Depression Scale.

* *P* < .05.

## 4. Discussion

### 4.1. Nursing programs based on action research can improve compliance in RA patients on long-term hormone therapy

RA is a chronic, progressive disease. There is currently no specific treatment plan. Patients need to take medication, control their diet, and perform functional exercises over a long period to control and delay the progression of the disease. Therefore, patients need to have a high level of compliance to complete the above. Many studies have shown that the factors influencing compliance are related to multiple factors, such as the patient, the disease, and the healthcare professional.^[17,18]^ Compliance is poor in people with diseases that require long-term rehabilitation treatment.^[19]^ Related studies have also shown that 35.9% of RA patients have poor medication compliance due to factors such as health literacy, disease course, and attention to the disease.^[20]^ Therefore, specific interventions are needed to improve rehabilitation and medication compliance in patients on long-term hormone therapy for RA. The results of this study showed that the scores of patients in the intervention group in terms of medication compliance, dietary compliance, and rehabilitation compliance were higher than those in the control group, indicating that the nursing program based on action research can improve the treatment compliance of patients on long-term hormone therapy for RA, which is consistent with the results of many studies.^[21–23]^ Notably, the between-group difference in total compliance score reached 29.63 points, with a Cohen *d* effect size of 8.35, indicating a very large effect. This is because the nursing program based on action research can be patient-centered, fully consider the patient’s ability, and work with the patient to solve a problem together, and through the cycle of problem discovery, planning, action, effect observation, reflection, and replanning, it can continuously and dynamically intervene with patients and improve the quality of clinical practice. Treatment compliance can be controlled by the patients themselves, which means that their subjective initiative must be given full scope. Researchers take a secondary role in meeting patients’ needs for disease-related knowledge and helping them to overcome misconceptions. In this study, firstly, during the acute phase of the disease, patients were treated for symptoms to meet their physiological needs, and to a certain extent, patients’ trust was gained so that they were willing to cooperate with the treatment; secondly, during the remission phase of the disease, patients were taught about the disease, and various forms were used to enable patients to master the relevant knowledge about RA and improve their health awareness. Finally, in the stable period of the disease, by holding a family symposium and setting up a WeChat communication group, a bridge for mutual learning and interaction was provided for the patients, and the supervisory role of family members and the role of the patient’s role model were played, which effectively enhanced the patients’ willingness and confidence in the treatment and enabled them to comply with the hormone treatment and rehabilitation exercises. In addition, through the power of a multidisciplinary team, personalized treatment plans are developed for patients based on their specific conditions, other than the disease, such as education level, personal income, etc, and health education methods that are easy for patients to accept and implement are used. These can increase patients’ subjective initiative and help turn plans into effective actions, thereby improving patients’ compliance with treatment.

### 4.2. Action research-based nursing programs can control increases in fasting and 2-hour postprandial glucose in RA patients on long-term hormone therapy

Glucocorticoids have become the first-line treatment for RA patients because of their potent anti-inflammatory and anti-allergic effects. Glucocorticoids are a class of steroid hormones secreted by the *zona fasciculata* of the adrenal cortex. They regulate the biosynthesis and metabolism of sugars, fats, and proteins. They can effectively promote protein metabolism and reduce the body’s use of glucose, causing the patient’s blood sugar to rise and increasing the risk of insulin resistance.^[23]^ Therefore, during hormone treatment of RA patients, the rise in blood glucose must be strictly controlled to avoid the occurrence of hormone-induced diabetes. The results of this study showed that the nursing program based on the action research method can control the fasting blood glucose and 2-hour postprandial blood glucose rise in RA patients receiving long-term hormone therapy. The intervention group demonstrated significantly better blood glucose control than the control group, with the difference being both statistically and clinically significant. This result is consistent with the findings of Zhang et al.^[24]^ The superior effect may be attributed to the comprehensive “monitoring-stratification-feedback-adjustment” closed-loop management model implemented in this study. This may be related to the fact that the nursing program based on the action research method proactively prioritized blood glucose management in the first round of action research, implementing hierarchical management from the outset. A 24-hour blood glucose curve was drawn for each patient within 24 hours of admission, providing the basis for individualized management and preventing sharp increases in fasting and postprandial blood glucose. This may be related to the fact that the nursing program based on the action research method proactively prioritized blood glucose management in the first round of action research, implementing hierarchical management from the outset. A 24-hour blood glucose curve was drawn for each patient within 24 hours of admission, providing the basis for individualized management and preventing sharp increases in fasting and postprandial blood glucose. At the same time, in the second round of action, the knowledge of blood glucose management was incorporated into the “Health Manual for Rheumatoid Arthritis Patients” in plain language, so that patients had a preliminary understanding of blood glucose management. At the same time, the relevant content of blood glucose stratification management was explained again in the special lecture, which further reinforced the importance of blood glucose management, taught patients the skills of blood glucose management, ensured the effectiveness of blood glucose management, and thus controlled the rise in blood glucose in patients.

### 4.3. Nursing programs based on action research can reduce joint pain and improve joint mobility in people with RA on long-term hormone therapy

At present, the pathogenesis of RA is not fully understood, and there is no specific cure. Therefore, symptom management, maintenance of joint function, and reduction of complications have become the primary demands and treatment goals of RA patients.^[25–27]^ Therefore, how to manage the pain symptoms of RA patients, reduce patients’ pain, and promote the recovery of joint function has become an urgent problem to be solved. The results of this study showed that the intervention program developed through action research can reduce the degree of joint pain in RA patients receiving long-term hormone therapy and increase the range of motion of the patients’ joints. The reduction in VAS score in the intervention group (mean decrease 3.97 points) corresponded to a large effect size (Cohen *d* = 2.11), which is markedly higher than the effect of conventional care reported by Jin et al^[26]^ (approximately 1.5-point reduction, Cohen *d* = 0.35). This highlights the superiority of the multidimensional intervention combining education, active exercise, and social support. The reason for the analysis may be that, based on the theory of action research, during the acute attack of the disease, a plan for pain management and functional exercise is formulated, pain management is strictly implemented according to the plan, and patients are guided to perform rehabilitation exercises. By educating patients on pain-related content, the method of pain assessment, the nature of pain, and the method of pain care are explained to improve the patient’s knowledge of pain; different intervention measures are taken for different degrees of pain to relieve the patient’s pain symptoms. By guiding patients to perform different types of rehabilitation exercises, patients can accept rehabilitation exercises. In addition, through the second round of observation and reflection, it was found that patients were less willing to do rehabilitation exercises. Through the third round of planning and action, measures such as family supervision and communication with patients were taken to increase patients’ willingness to do rehabilitation exercises, which played a positive role in improving joint function.

### 4.4. Action research-based nursing program can reduce anxiety and depression in RA patients on long-term hormone therapy

Patients with RA undergoing long-term hormone therapy find themselves in a perpetual state of managing the disease, necessitating ongoing treatment. This dual burden, involving concerns about the adverse effects of hormone therapy and the persistent discomfort from the disease itself, exacerbates anxiety and depression among these patients.^[28]^ Such negative emotional states not only hinder the therapeutic response but also significantly degrade the quality of life.^[29]^ Consequently, clinical interventions aimed at mitigating anxiety and depression are imperative. The findings of this study indicate that a nursing program based on action research effectively alleviates anxiety and depression in RA patients undergoing long-term hormone therapy, corroborating the results reported by Yan et al.^[30]^ The intervention achieved substantial improvements in both anxiety and depression scores, outperforming the effects typically observed with routine psychological care. This advantage likely stems from the integrated “symptom relief-knowledge empowerment-social support” pathway that addresses the specific stressors of RA patients, including hormone concerns, chronic course, and functional limitations. The primary contributors to anxiety and depression in these patients include physical symptoms such as pain, insufficient disease comprehension, concerns regarding medication side effects, and inadequate understanding of rehabilitation exercises. The action research-based nursing program developed in this study addresses these issues comprehensively. By integrating relevant knowledge lectures and health education, the program enhances patients’ understanding of the disease and medication management. In addition, by guiding patients through rehabilitation exercises, the program improves their proficiency and adherence to these exercises, fostering a correct perception of the disease. Consistent engagement in rehabilitation exercises ameliorates physical symptoms and restores joint function, thereby reducing anxiety and depression among patients.

### 4.5. Limitations

This study has several limitations. First, it was conducted at a single center with a relatively small sample size, which may limit the generalizability of the findings. Second, the intervention period was only 4 weeks, and no long-term follow-up was conducted to assess the sustainability of the observed effects. Future multicenter studies with larger sample sizes and extended follow-up periods are warranted to validate these results and evaluate the long-term impact of the action research-based nursing program.

## 5. Conclusion

The action research-based nursing program significantly improved the management and outcomes for RA patients undergoing long-term hormone therapy. The intervention group, which received a structured and multidisciplinary care plan, demonstrated better compliance, reduced pain symptoms, and enhanced joint mobility compared to the control group. In addition, the intervention effectively controlled blood glucose levels and alleviated anxiety and depression among patients. These findings suggest that the action research-based nursing approach is an effective strategy for managing the complex needs of RA patients on long-term hormone therapy, providing a valuable framework for clinical practice and highlighting the importance of continuous, dynamic, and patient-centered care. This model of care provides a practical reference for improving the quality of life and clinical outcomes for RA patients.

## Acknowledgments

The authors express their gratitude to all participants involved in this study.

## Author contributions

**Conceptualization:** Lin Sun, Chunfang Gu.

**Formal analysis:** Lin Sun.

**Methodology:** Lin Sun, Yue Zhang, Hai Huang, Chunfang Gu.

**Writing – original draft:** Lin Sun.

**Writing – review & editing:** Lin Sun, Chunfang Gu.

**Project administration:** Chunfang Gu.
